# Uvulopalatopharyngoplasty and barbed reposition pharyngoplasty with and without hyoid suspension for obstructive sleep apnea hypopnea syndrome: A comparison of long-term functional results

**DOI:** 10.17305/bjbms.2020.4724

**Published:** 2021-06

**Authors:** Antonio Minni, Fabrizio Cialente, Massimo Ralli, Andrea Colizza, Quirino Lai, Angelo Placentino, Melania Franco, Valeria Rossetti, Marco de Vincentiis

**Affiliations:** 1 Department of Sense Organs, Sapienza University of Rome, Rome, Italy; 2 Hepato-bilio-pancreatic and Liver Transplant Unit, Department of Surgery, Sapienza University of Rome, Rome, Italy; 3 Azienda Ospedaliera Ca’ Grande Niguarda, Milan, Italy; 4 Department of Oral and Maxillofacial Sciences, Sapienza University of Rome, Rome, Italy

**Keywords:** Obstructive sleep apnea hypopnea syndrome, uvulopalatopharyngoplasty, barbed reposition pharyngoplasty, surgery

## Abstract

Obstructive sleep apnea hypopnea syndrome (OSAHS) is a common condition; when conservative approaches are not effective, surgical techniques aimed at reducing the airway obstruction effect are used. This retrospective study aimed at comparing the functional outcomes in patients with OSAHS undergoing uvulopalatopharyngoplasty (UPPP) according to Fairbanks and barbed reposition pharyngoplasty (BRP) according to Mantovani, with or without hyoid suspension (HS). One-hundred twenty-two consecutive OSAHS patients who underwent surgical treatment were included in the study. Patients were divided into 4 groups; all patients underwent preoperative and postoperative polysomnography (PSG) with apnea/hypopnea index (AHI) and oxygen desaturation index (ODI) evaluation, and Epworth Sleepiness Scale (ESS) evaluation. The results were analyzed according to the different surgical procedures in relation to the preoperative PSG and anthropometric data. A significant reduction was observed at 18-month follow-up for patients in BRP group for body mass index (*p* = 0.004), ESS (*p* < 0.0001), ODI (*p* < 0.0001), and AHI (*p* < 0.0001). Risk factors for poor postoperative AHI reduction were evaluated; preoperative AHI was the strongest independent protective factor, while preoperative ODI was the strongest risk factor. The association of HS with UPPP or BRP showed significant results in terms of higher postoperative AHI reduction only when associated to UPPP (*p* < 0.0001). This study showed that the BRP technique was more effective compared to UPPP for patients with OSAHS. The association of HS showed greater benefits in UPPP compared to BRP. Randomized prospective trials with longer follow-up are necessary to confirm our results and formulate a more accurate indication of the optimal therapeutic strategy.

## INTRODUCTION

Obstructive sleep apnea hypopnea syndrome (OSAHS) is a common condition affecting 23.4% of women and 49.7% of men over 40 years old [1,2]. The risk factors include age, male gender, cigarette smoking, obesity, and abnormal facial anatomy [3,4]. The clinical symptoms are excessive daytime sleepiness [5-7], morning headache [8], decrease of cognitive performance [9,10], sexual dysfunction [11], decreased quality of life [12-16], and increased cardiovascular risk [17-20].

The main pathological event of OSAHS is the collapse of the upper airways that may occur at the same time at different levels, such as nasal, retropalatal and/or retrobasilingual and/or laryngeal [21]. However, the most frequent site of collapse is the soft palate, followed by the pharyngeal walls, base of the tongue and palatine tonsils. The larynx, and especially the epiglottis, is less involved [22].

The primary management of OSAHS relies on conservative approaches such as improved sleep hygiene, weight loss, use of dental splints, and continuous positive airway pressure (CPAP) treatment [23-27]. CPAP has been first used to maintain the patency of the upper airways during sleep by Sullivan et al. in 1981 [28]; their results were confirmed by several follow-up studies and CPAP now represents the gold standard for OSAHS treatment. However, nearly 40% of patients show an intolerance to CPAP machine and require alternative treatments, including surgery [29,30].

Surgical techniques for OSAHS aim at reducing the airway obstruction effect due to the excessive bulk of soft tissues lining the rhino-oro-hypopharynx, and they may be performed as single or combined procedures and traditional or robot-assisted, depending on patient conditions [31-36]. The most common surgical procedure for OSAHS is uvulopalatopharyngoplasty (UPPP), first described by Fujita in 1984 [37] and subsequently standardized by Fairbanks in 1999 [38]. UPPP is used to treat the retropalatal region; however, it only treats obstruction in the soft palate, while it does not address the collapse at different levels. Simple UPPP as a treatment of OSAHS has a success rate that ranges between 16% and 83% [39-41]. Furthermore, the recurrence rate of OSAHS at 10 years is as high as 40%, especially in obese patients [42,43]. To overcome these limits of UPPP, Mantovani et al. [44] proposed in 2012 a new surgical technique, the barbed reposition pharyngoplasty (BRP), which laterally and anteriorly displaces the posterior pillar to enlarge the oropharyngeal inlet and the retropalatal space [22].

This retrospective study aimed at comparing the functional outcomes in patients with OSAHS undergoing UPPP according to Fairbanks and BRP according to Mantovani, with or without hyoid suspension (HS).

## MATERIALS AND METHODS

One-hundred twenty-two consecutive patients with a definitive diagnosis of OSAHS who underwent surgical treatment between January 2015 and December 2018 in the Otolaryngology unit of our University hospital were included in this retrospective study. All patients signed a written informed consent; the procedures were performed in accordance with the standards of the ethics committee on human experimentation of our University Department that specifically approved this study, and in accordance with the Helsinki Declaration.

The inclusion criteria were age between 25 and 75 years, body mass index (BMI) >15 and <35, any degree of tonsillar volume, apnea-hypopnea index (AHI) >15, and failure of preoperative CPAP treatment.

The exclusion criteria were patients with severe medical conditions, patients with craniofacial anomalies that had affected airways, patients with limited mouth opening, prior airway surgery, and patients with the American Society of Anesthesiologists (ASA) physical status score >2.

All patients underwent preoperative otolaryngology clinical evaluation, endoscopic examination with Mueller maneuver, polysomnography (PSG) with AHI and oxygen desaturation index (ODI) evaluation, and Epworth sleepiness scale (ESS) evaluation. Clinical information, including age and gender, smoking history and comorbidities, were collected at the first visit for each patient.

Surgery was performed by the same surgeon using UPPP according to Fairbanks or the BRP technique according to Mantovani. The two procedures were performed alone or combined with HS, a hypopharyngeal procedure that allows lateral traction of the hypopharynx and moderate advancement of the base of the tongue.

Patients were subsequently divided into two groups based on the surgical procedure, group A: UPPP; group B: BRP.

Otolaryngology examination, PSG, and ESS were repeated in all patients 18 months after surgery. At follow-up visit, patients were classified based on PSG results as *recovery* (AHI<5, ESS<10, both reduced >50%), *success* (AHI<20, ESS <10, both reduced >50%), and *failure* (AHI>20, ESS>10, both reduced <50%).

Results were analyzed according to the different surgical procedures in relation to the preoperative PSG (AHI, ODI) and anthropometric (BMI) data.

### Statistical analysis

Continuous variables were reported as medians and interquartile ranges (IQR). Dummy variables were reported as numbers and percentages. Mann–Whitney U test and Fisher’s exact test were used for comparisons of categorical and continuous variables. A multivariable logistic regression model was built with the intent to identify the risk factors for poor postoperative decreasing of AHI score. We defined as poor a decrease >50% after surgery, according to the definition by Rashwan et al. [45]. Seven different covariates were initially investigated in the model: age, gender, BRP as a surgical approach, and preoperative values of BMI, ESS, ODI, and AHI. A backward Wald method was used for the construction of the final model. Odds ratios (OR), standard errors (SE), and 95% confidence intervals (95% CI) were reported. Model fitting was tested adopting the Hosmer–Lemeshow test. A *p* < 0.05 was defined for significance. We used IBM SPSS Statistics for Windows, Version 24.0 (IBM Corp., Armonk, NY, USA).

## RESULTS

### Comparison between UPPP and BRP groups

**FIGURE 1:**
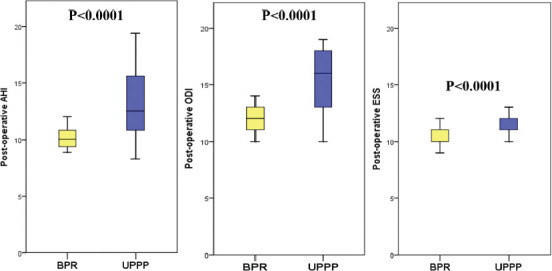
Postoperative values of apnea-hypopnea index (AHI), oxygen desaturation index (ODI), and Epworth sleepiness scale (ESS) in the two cohorts of patients treated with barbed reposition pharyngoplasty (BRP) versus uvulopalatopharyngoplasty (UPPP) technique.

Demographics and postoperative course of patients that underwent UPPP and BRP are reported in Table 1. No significant differences were found between the two groups for age (*p* = 0.5), gender (*p* = 0.1), and contemporaneous HS procedure (*p* = 1.0). Similarly, preoperative ESS (*p* = 0.5) and ODI (*p* = 0.3) did not significantly differ. BRP cases had a higher preoperative BMI value compared to subjects in the UPPP group (*p* < 0.0001). Interestingly, all the preoperative variables significantly improved postoperatively in the BRP group. In this group, a significant reduction was observed for median delta BMI (*p* = 0.004), median delta ESS (*p* < 0.0001), ODI (*p* < 0.0001), and AHI (*p* < 0.0001). Only one patient (2.4%) in the BRP group showed a postoperative AHI reduction <50%, while 36 (45.0%) cases were reported in the UPPP group (*p* < 0.0001). Postoperative values of AHI, ODI, and ESS in the two groups are shown in Figure 1.

**TABLE 1:**
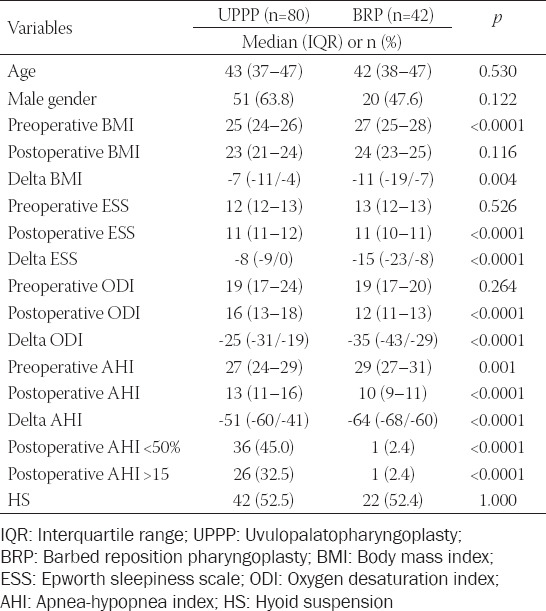
Comparison between UPPP and BRP groups

### Risk factors for poor postoperative AHI reduction

A multivariable logistic regression model was constructed aimed to identify the prognostic variables for poor postoperative AHI reduction (defined as <50%), and three independent covariates were identified. The results are shown in Table 2.

**TABLE 2:**
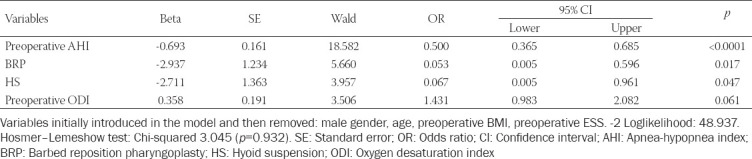
Multivariable logistic regression analysis for the risk of postsurgical AHI reduction <50%. Backward Wald method was adopted

Preoperative AHI value was the strongest independent protective factor, with an OR = 0.5 (95% CI = 0.4–0.7; *p* < 0.0001), indicating that the higher the preoperative AHI value, the lower the risk of experiencing a poor postoperative AHI decrease, with a 50% risk reduction for each unit increase in the preoperative AHI values. In addition, the surgical procedures BRP and HS were independent protective factors, with ORs = 0.05 (95% CI = 0.005–0.6; *p* = 0.02) and 0.07 (95% CI = 0.005–0.96; *p* = 0.047), respectively. In other terms, undergoing a BRP or a HS corresponded to a 95% and a 93% reduction in the risk of experiencing a postoperative poor AHI reduction value, respectively.

The preoperative ODI value was a significant risk factor for poor postoperative AHI reduction (OR = 1.9, 95% CI = 1.4–2.5; *p* < 0.0001); this indicates that the higher the preoperative ODI value, the higher the risk of poor postoperative AHI reduction.

### Role of HS in combination with UPPP and BRP

According to the results observed in the multivariable model, in which HS had a positive role in reducing the risk of poor postoperative AHI reduction, a sub-analysis was performed aimed at identifying the combinatory effect of HS in the case of UPPP or BRP (Table 3). Interestingly, a substantial difference was observed when the sub-group of UPPP patients (n = 38) was compared with the other groups regarding postoperative AHI decreasing (*p* < 0.0001). The vast majority of cases treated only with UPPP showed a poor AHI decrease (n = 30; 78.9%), while the patients requiring a combinatory UPPP+HS treatment (n = 42) showed intermediate results (n = 6, 14.3%). Only one subject of poor AHI decrease was observed among the subjects treated with BRP (n = 20) or BRP+HS (n = 22).

**TABLE 3:**
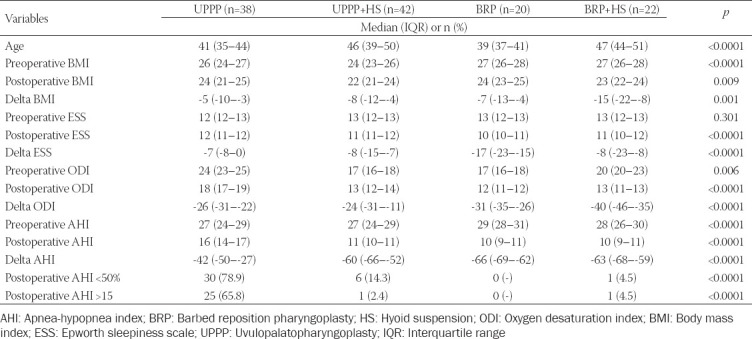
Comparison among the cohorts according to the different surgical strategy adopted

## DISCUSSION

In the present study, we compared the functional outcomes of patients with OSAHS undergoing UPPP according to Fairbanks and BRP according to Mantovani. Our results show that BRP is more effective than UPPP; in fact, considering a similar value of the preoperatory AHI index, we found a greater decrease of postoperative AHI with the BRP technique compared to UPPP (10 vs. 16). A possible explanation is that BRP guarantees, compared to UPPP, a greater and more stable retraction of the pharyngeal soft tissue due to the latero-lateral traction and the anchorage to the pterygomandibular raphe, an enlargement of the anteroposterior space and better preservation of the mucosa and muscle tissue.

Our results also show that the efficacy of BRP in terms of AHI is not improved by HS. The UPPP surgical procedure, instead, showed a greater efficacy if performed with HS. The execution of HS reduces the latero-lateral hypopharyngeal collapse with the result of increasing the transverse diameters of the upper pharynx, optimizing in this way the action of the UPPP procedure. Contrarily, BRP guarantees an effective retropalatal enlargement without the necessity of HS. This is consistent with the results of previous studies [46].

Although some aspects of the pathophysiology of OSAS are still unknown, it has been widely accepted that pharyngeal obstruction during the apnea/hypopnea episodes derives from a complex set of anatomical and functional factors. OSAHS patients often present an obstruction at multiple levels of the upper airways and the sole execution of UPPP is frequently inadequate [41]. The most recent acquisitions in OSAHS surgery recommend to expand and stabilize the pharyngeal space; often, a satisfactory outcome is achieved with the combination of UPPP with other nasopharyngeal or oropharyngeal procedures. Riley et al. [47] proposed phase I multiple-level surgery for OSAHS patients using genioglossus advancement (GA) combined with HS. The development of new diagnostic procedures such as drug-induced sleep endoscopy (DISE) has allowed a better preoperative identification of the individual contribution of the different sites of obstruction. Nowadays, DISE is a fundamental procedure to plan a targeted and personalized surgical approach for each patient. At this regard, DISE allows to exclude from the traditional surgical procedures patients with OSAHS that originate from the collapse of the epiglottis due to hypertrophy of the tongue base, in which the reduction of the base of the tongue with robotic surgery has been shown to be more effective [48]. The advantages of robotic surgery are a good anatomical exposure of the surgical field, control of vascular and nervous structures, reduction of time of surgery, better aesthetic result, and improvement of quality of life.

The main limitation of our study is the relatively short follow-up (18 months); further studies with longer follow-up are necessary to confirm our findings.

## CONCLUSION

This retrospective study showed that the BRP technique according to Mantovani was more effective, in the short term, compared to the classic UPPP technique of Fairbanks for patients with OSAHS. The combined use of HS showed greater benefits in UPPP compared to BRP. Randomized prospective trials with longer follow-up are necessary to confirm our results and formulate a more accurate indication of the optimal therapeutic strategy.
